# Enhancement of Nucleoside Production in* Hirsutella sinensis* Based on Biosynthetic Pathway Analysis

**DOI:** 10.1155/2017/2520347

**Published:** 2017-11-29

**Authors:** Zhi-Qiang Liu, Bo Zhang, Shan Lin, Peter James Baker, Mao-Sheng Chen, Ya-Ping Xue, Hui Wu, Feng Xu, Shui-Jin Yuan, Yi Teng, Ling-Fang Wu, Yu-Guo Zheng

**Affiliations:** ^1^Key Laboratory of Bioorganic Synthesis of Zhejiang Province, College of Biotechnology and Bioengineering, Zhejiang University of Technology, Hangzhou 310014, China; ^2^Department of Nephrology, Zhejiang Provincial People's Hospital, Hangzhou 310014, China; ^3^East China Pharmaceutical Group Limited Co., Ltd, Hangzhou 311000, China

## Abstract

To enhance nucleoside production in* Hirsutella sinensis*, the biosynthetic pathways of purine and pyrimidine nucleosides were constructed and verified. The differential expression analysis showed that* purine nucleoside phosphorylase*,* inosine monophosphate dehydrogenase,* and* guanosine monophosphate synthase* genes involved in purine nucleotide biosynthesis were significantly upregulated 16.56-fold, 8-fold, and 5.43-fold, respectively. Moreover,* dihydroorotate dehydrogenase*,* uridine nucleosidase*,* uridine/cytidine monophosphate kinase,* and* inosine triphosphate pyrophosphatase* genes participating in pyrimidine nucleoside biosynthesis were upregulated 4.53-fold, 10.63-fold, 4.26-fold, and 5.98-fold, respectively. To enhance the nucleoside production, precursors for synthesis of nucleosides were added based on the analysis of biosynthetic pathways. Uridine and cytidine contents, respectively, reached 5.04 mg/g and 3.54 mg/g when adding 2 mg/mL of ribose, resulting in an increase of 28.6% and 296% compared with the control, respectively. Meanwhile, uridine and cytidine contents, respectively, reached 10.83 mg/g 2.12 mg/g when adding 0.3 mg/mL of uracil, leading to an increase of 176.3% and 137.1%, respectively. This report indicated that fermentation regulation was an effective way to enhance the nucleoside production in* H. sinensis* based on biosynthetic pathway analysis.

## 1. Introduction

*Ophiocordyceps sinensis* (renamed from* Cordyceps sinensis*) has been well known and used as a traditional medicine or healthful food in China and is generally found on the Tibetan Plateau at high altitudes ranging from 3,600 to 5,400 m [1, 2]. Previous studies have revealed that* O. sinensis* and its anamorph possess a variety of biologically effective compounds with extensive pharmacological effects [3]. Due to the unique medicinal value, the natural* O. sinensis *is facing high demand and insufficient supply. It is reported that mycelia of the* Cordyceps* species produced by fermentation have become a feasible and sustainable means for producing the medicinal fungus and bioactive compounds [4].* Hirsutella sinensis* is currently considered as the only correct anamorph of* O. sinensis* based on its morphological and phylogenetic characteristics [5]. Bioactive compounds from mycelia of* H. sinensis* by submerged cultivation have been reported to have similar clinical efficacy and less associated toxicity compared with wild* O. sinensis*, indicating that the submerged cultivation of* H. sinensis* has the trend of gradually replacing the* O. sinensis* in the market [6, 7]. Therefore, with the increasing interest in* Cordyceps* both for the mycology and the pharmacology, it is more and more urgent to investigate the application of* H. sinensis*.

Nucleosides including purine and pyrimidine nucleosides are reported to possess many interesting forms of biological and pharmacological activity, such as anti-infective activity [8], antiviral activity [9], anticancer activity [10], antioxidation activity [11], and immunological stimulation [12], which implies a need for large-scale production of nucleosides. The concentration of nucleosides is also an important standard for quality control of* O. sinensis*. To date, more than 10 nucleosides, nucleobases, and their related compounds including adenine, adenosine, cytosine, cytidine, uridine, guanine, guanosine, hypoxanthine, vernine, thymidine, cordycepin, and 2′-deoxyuridine have been isolated from* O. sinensis* and identified [13]. Currently, three extraction methods, namely, organic solvent (methanol) pressurized liquid extraction (OSPLE), boiling water extraction (BWE), and ambient temperature water extraction (ATWE), are commonly applied for the extraction of nucleosides from natural and artificially cultured* O. sinensis* [14]. The effects of extraction methods on the quantification of nucleosides in different samples of* O. sinensis* are varied [15]. A rapid ultraperformance liquid chromatography method has been developed for the simultaneous determination of nucleoside from cultured* Jiangxi O. sinensis*, and cytosine (98.9 *μ*g/g), uracil (301.3 *μ*g/g), cytidine (832.8 *μ*g/g), uridine (3214.4 *μ*g/g), and adenine (566.2 *μ*g/g) were determined [16]. Moreover, an ion-pairing reversed-phase liquid chromatography/mass spectrometry procedure was generated for the determination of nucleosides from* O. sinensis*, and the components of nucleosides including adenosine (0.55–8.85 g/mL), uridine (0.33–10.50 g/mL), cytidine (0.48–15.30 g/mL), thymidine (0.20–6.30 g/mL), guanine (0.26–4.20 g/mL), uracil (0.38–12.15 g/mL), cytosine (0.39–12.45 g/mL), and thymine (0.26–8.25 g/mL) were determined [17].

Previous studies have investigated the enhancement of the production of target products based on biosynthetic pathway analysis [18–20]. The cordycepin and cordycepic acid production from* H. sinensis* was improved based on biosynthetic pathway analysis and differential expression analysis [18]. The production of* n*-alkanes in* Escherichia coli* was significantly enhanced through spatial organization of enzymes involved in its biosynthetic pathway [19]. Metabolic engineering of the early non-mevalonate terpenoid pathway in* E. coli* was carried out to increase the supply of prenyl pyrophosphates as precursors for carotenoid production, and the carotenoid content reached 1.6 mg/g [20]. These studies indicated that it is feasible to enhance nucleoside production by analyzing and regulating their biosynthetic pathways. To date, there are few reports of enhancing the nucleoside production in* H. sinensis*. Our previous studies in transcriptome sequencing and analysis discovered the genes and enzymes involved in biosynthetic pathways, which benefited the investigation of biosynthetic pathway analysis for enhancing their production [21].

In this study, the biosynthetic pathways of purine and pyrimidine nucleosides in* H. sinensis* were constructed and verified. The differential expression analysis of the genes that encode the enzymes involved in the biosynthetic pathways for purine and pyrimidine nucleosides was performed, and the significantly upregulated genes corresponding to important enzymes were determined. Based on the analysis of biosynthetic pathways and gene differential expressions, fermentation regulation of* H. sinensis* was carried out, and the positive effects on nucleoside production were obtained. The fermentation regulation was observed to be efficient in enhancing the nucleoside production in* H. sinensis*.

## 2. Materials and Methods

### 2.1. Materials and Chemicals

*H. sinensis* L0106 was isolated from wild* O. sinensis* samples that were gingerly collected from Qinghai-Tibet Plateau in Qinghai Province.* H. sinensis* L0106 has been deposited at the China Center for Type Culture Collection (Wuhan, China) with accession number CCTCC M2011278. Subsequently, the transcriptome of* H. sinensis* was sequenced and the reads obtained were further assembled into Unigenes, and then BLASTx alignment (*e *< 0.00001) between protein databases and Unigenes was performed [21].* E. coli *JM109 was selected as host for plasmid pMD18-T transformation (Invitrogen, Carlsbad, CA, USA), and* E. coli* BL21 (DE3) (Invitrogen) was selected as the host for expression of pET28a (Invitrogen). Lysogeny broth (LB) medium (10 g/L tryptone, 5 g/L yeast extract, and 10 g/L NaCl) at 37°C with shaking (200 rpm) was used for the growth of* E. coli* transformants. Ampicillin, kanamycin, isopropyl-*β*-D-thiogalactopyranoside (IPTG), adenosine, vernine, cytidine, uridine, and thymidine standard substances were purchased from Sigma Chemical Co. (St. Louis, MO, USA).

### 2.2. Submerged Fermentation of* H. sinensis*

Slant agar medium for cultivation of* H. sinensis* consisted of powdered silkworm pupae (1.5%), corn flour (1%), bran (1.5%), glucose (1.5%), dextrin (0.5%), yeast extract (0.5%), tryptone (1%), MgSO_4_ (0.01%), KH_2_PO_4_ (0.02%), and agar (2%). Powdered silkworm pupae, corn flour, and bran were first prepared and liquefied at 121°C for 20 min, and then the liquefied medium obtained was used to dissolve other medium components.* H. sinensis* L0106 was cultured on solid slant medium at 16°C for approximately 30 days, and then the colonies with 2 cm diameter were formed and 5 of them were transferred to seed medium using an inoculating shovel at 16°C for 20 days on a rotary shaker at 150 rpm. Furthermore, 5% seed medium was inoculated with submerged fermentation medium in 500 mL shake flasks, and mycelia were asexually reproduced and harvested for approximately 10 days. Submerged fermentation medium and seed medium had the same components as slant medium without agar.

### 2.3. Screening and Verifying Biosynthetic Genes of Purine and Pyrimidine Nucleosides

To study the biosynthetic pathway of purine nucleotides in* H. sinensis*, the purine metabolism pathway (map00230) in* H. sinensis* was constructed based on the KEGG annotation of the* H. sinensis* transcriptome. Meanwhile, based on the pyrimidine metabolism pathway (map00240), which was involved in the KEGG metabolic pathway annotation of the* H. sinensis* transcriptome, the biosynthetic pathway of pyrimidine nucleosides in* H. sinensis* was constructed.

The functional genes and enzymes involved in the biosynthetic pathways of purine and pyrimidine nucleosides in* H. sinensis *were verified by gene cloning and protein expression [22–24]. PCR was performed to verify the genes encoding the enzymes involved in the biosynthetic pathways of purine and pyrimidine nucleosides. Primers were designed based on the predicted open reading frame (ORF) sequences of target genes (Tables S1 and S2, Supporting Information), and then the genes encoding the enzymes were subcloned to the pMD18-T vectors. Subsequently, the recombinant plasmids were successfully transformed to* E. coli *JM109 competent cells. The recombinant* E. coli *JM109 was cultivated in the shaker at 37°C, and the recombinant plasmids were extracted using a plasmid extraction kit (Qiagen, Hilden, Germany). After being sequenced, the target genes were subcloned into pET28a vectors, and then the recombinant plasmids were further transferred to* E. coli *BL21 competent cells. After inducing by IPTG, the genes were expressed in* E. coli *BL21, and then the expressed proteins were detected by 12% sodium dodecyl sulfate polyacrylamide gel electrophoresis (SDS-PAGE).

### 2.4. Real-Time PCR

Three different cDNA libraries from mycelia of* H. sinensis *cultured by submerged fermentation for 3 days (growth period), 6 days (before stable period), and 9 days (stable period) were prepared by a cDNA library construction kit (TaKaRa, Dalian, China) according to the manufacturer's protocols. Meanwhile, primers for real-time PCR were designed by the Primer Express tool (Applied Biosystems, Foster City, CA, USA), and the 18S rRNA gene was selected as the internal control. Real-time PCR mixture (10 *μ*L) was prepared and consisted of 1 *μ*L of cDNA from 3-day, 6-day, and 9-day samples, respectively, 5 *μ*L of SYBR Green PCR Master Mix (2x) (Promega), and 0.5 *μ*L (100 *μ*mol/L) of each forward and reverse primer. Real-time PCR analyses were performed three times with independent RNA samples according to the temperature-time profile as follows: denaturation of 95°C for 2 min, 40 cycles of 95°C for 15 sec, 60°C for 1 min.

### 2.5. Differential Expression Analysis of Biosynthetic Genes

The 2^−ΔΔCt^ method was applied to calculate the relative expression levels by comparing the cycle thresholds (CTs) of the target genes with the CTs of the 18S rRNA gene. Differences in relative transcript expression levels of different samples were compared at the *p* < 0.05 level among different culture periods of* H. sinensis* using Student's* t*-test. Eight primers of the gene-encoding enzymes involved in purine nucleotide biosynthesis (Table S3, Supporting Information), as well as thirteen primers of the gene-encoding enzymes involved in pyrimidine nucleoside biosynthesis (Table S4, Supporting Information), were used to amplify the target products. Differential expression of up- or downregulated genes detected in the microarray experiments was determined by real-time PCR. The significantly upregulated genes were determined and selected as important genes encoding important enzymes involved in biosynthetic pathways of purine and pyrimidine nucleosides in* H. sinensis*.

### 2.6. Strategy of Fermentation Regulation Based on the Biosynthetic Pathway

In the biosynthetic pathway of pyrimidine nucleosides, ribose and uracil are the two products catalyzed by uridine nucleosidase. Certain concentrations of ribose and uracil may cause product inhibition of uridine nucleosidase and lead to the accumulation of uridine. To investigate the effects of addition of uracil on production of pyrimidine nucleosides, 0, 1.0, 2.0, 3.0, and 4.0 mg/mL ribose or 0, 0.3, 0.6, 1.2, and 1.9 mg/mL uracil were added to the 100 mL of the submerged fermentation medium of* H. sinensis* according to preliminary experiments.

### 2.7. Determination of Nucleoside Content

Mycelia of* H. sinensis* were harvested after fermentation and washed three times with ultrapure water and then placed in an oven at 60°C for drying to constant weight. The dried mycelia were weighed, pounded to powder, and sealed under refrigeration at 4°C for further use. To analyze nucleoside content, 0.50 g of the dried powder of* H. sinensis* mycelia was accurately weighed and extracted with 10 mL of a solution of water and ethyl alcohol (75 : 25, v/v), and then the mixture was placed in an ultrasonicator for 30 min and centrifuged at 12,000 rpm for 10 min. Supernatant was collected and the residue was reextracted twice. The extract obtained above was filtered through a 0.22 *μ*m filter and further analyzed. Assay of nucleoside content was carried out by high performance liquid chromatography (HPLC) according to a reported procedure with modifications [25]. The column temperature was maintained at 35°C. The standards or samples were separated using a gradient mobile phase consisting of ultrapure water (A) and methyl alcohol (B). The gradient conditions are 0–3 min, 15% B; 3.0–3.5 min, 15–25% B; 3.5–8.55 min, 24% B; 8.5–9.0 min, 24–35% B; 9.0–15.0 min, 35% B; 15.0–16.0 min, 35–85% B; 16.0–22.0 min, 85% B; 22.0–22.5 min, 85–15% B and 22.5–27.5 min, 15% B. The column was rinsed with 100% methyl alcohol for every ten runs. The flow rate was set at 1.0 mL/min. The peaks were detected at 260 nm and identified by comparing the retention times with the standard. A standard calibration curve was drawn by consecutively injecting different concentrations of standard adenosine, vernine, cytidine, uridine, and thymidine. The standard curves were prepared, and the linear regression equations are shown below:* Y*_1_ = 564.89*X*_1_ − 0.8929, *R*^2^ = 0.9998, where* Y*_1_ stands for the adenosine concentration of the standard solution and* X*_1_ stands for the peak area of adenosine;* Y*_2_ = 477.01*X*_2_ − 1.3681, *R*^2^ = 0.9951, where* Y*_2_ stands for the vernine concentration of the standard solution and* X*_2_ stands for the peak area of vernine;* Y*_3_ = 321.19*X*_3_ − 0.2774, *R*^2^ = 0.9997, where* Y*_3_ stands for the cytidine concentration of the standard solution and* X*_3_ stands for the peak area of cytidine;* Y*_4_ = 406.34*X*_4_ + 5.3884, *R*^2^ = 0.9951, where* Y*_4_ stands for the uridine concentration of the standard solution and* X*_4_ stands for the peak area of uridine; and* Y*_5_ = 386.27*X*_5_ − 0.1233, *R*^2^ = 0.9995, where* Y*_5_ stands for the thymidine concentration of the standard solution and* X*_5_ stands for the peak area of thymidine. The percentage of nucleoside extraction yield (mg/g) was calculated as the nucleoside content of the extraction divided by dried sample weight.

### 2.8. Statistical Analysis

In this study, all experiments were performed in triplicate if not specifically noted. The data were analyzed by the statistical software SPSS (version 8.0, IBM, Chicago). Student's* t*-test and the analysis of variance (ANOVA) test were performed (*p* < 0.05).

## 3. Results

### 3.1. Constructing Biosynthetic Pathways of Purine and Pyrimidine Nucleosides

**Figure 1:**
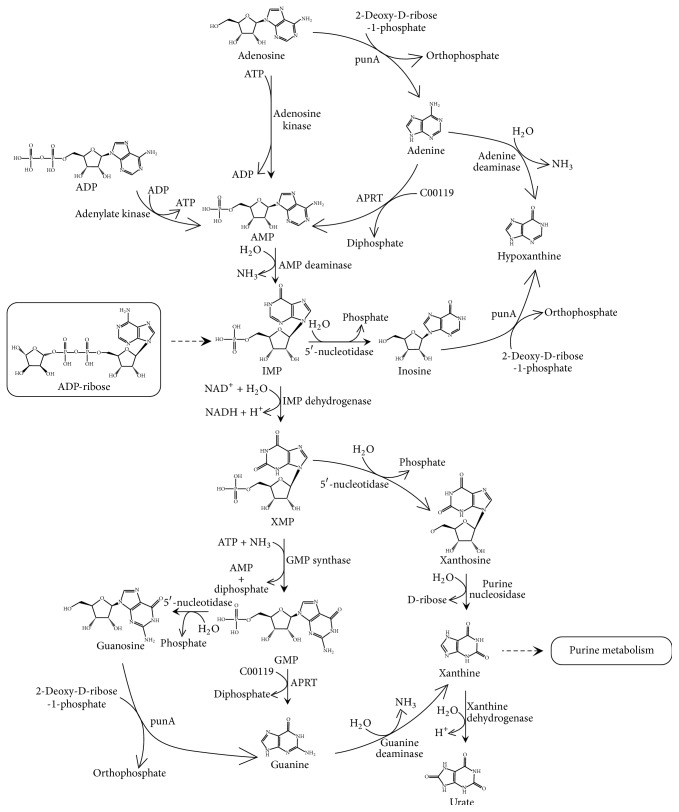
*Biosynthetic pathway for purine nucleotides in H. sinensis.* The biosynthetic pathway of purine nucleotides in* H. sinensis* was constructed based on KEGG annotation of* H. sinensis* transcriptome. The punA, adenosine kinase, APRT, AMP deaminase, IMP dehydrogenase, GMP synthase, and guanine deaminase involved in the biosynthetic pathway for purine nucleotides were annotated in the* H. sinensis* transcriptome.

The transcriptomes of* H. sinensis* in different cultivation periods (3 days, 6 days, and 9 days) were sequenced, the obtained reads were further assembled, and 20,822 Unigenes (All) were generated. For protein functional annotation, the Unigene sequences were searched using BLASTx against the protein databases [21]. From the analysis and annotation of* H. sinensis* transcriptome, most enzymes involved in biosynthetic pathways of purine and pyrimidine nucleosides were annotated (Tables S5 and S6, Supporting Information). Based on the annotated purine metabolic pathway (Figure S1, Supporting Information), the annotated enzymes were used to construct purine nucleotide biosynthesis, and a proposed biosynthetic pathway for purine nucleotides was obtained (Figure 1). In this biosynthetic pathway, purine nucleoside phosphorylase (punA) that converts adenosine to adenine, adenosine kinase that converts adenosine to adenosine 5′-monophosphate (AMP), adenine phosphoribosyltransferase (APRT) that converts adenine to AMP, AMP deaminase that converts AMP to IMP, inosine monophosphate (IMP) dehydrogenase that converts IMP to XMP, guanosine monophosphate (GMP) synthase that converts XMP to GMP, and guanine deaminase that converts GMP to guanine were, respectively, determined (Figure 1). Particularly, punA, adenosine kinase, APRT, AMP deaminase, IMP dehydrogenase, GMP synthase, and guanine deaminase that were involved in purine nucleotide biosynthesis were discovered in the annotation dataset of* H. sinensis*. Unigene14697_All was annotated to* AK*, Unigene1297_All was annotated to* adenosine kinase*, Unigene12674_All was annotated to* APRT*, Unigene13557_All and Unigene3652_All were annotated to* AMP deaminase*, Unigene6288_All and Unigene9455_All were annotated to* IMP dehydrogenase*, and Unigene11031_All, Unigene11950_All, and Unigene13696_All were annotated to* GMP synthase*. In addition, Unigene7330_All and Unigene9274_All were annotated to* guanine deaminase*.

**Figure 2:**
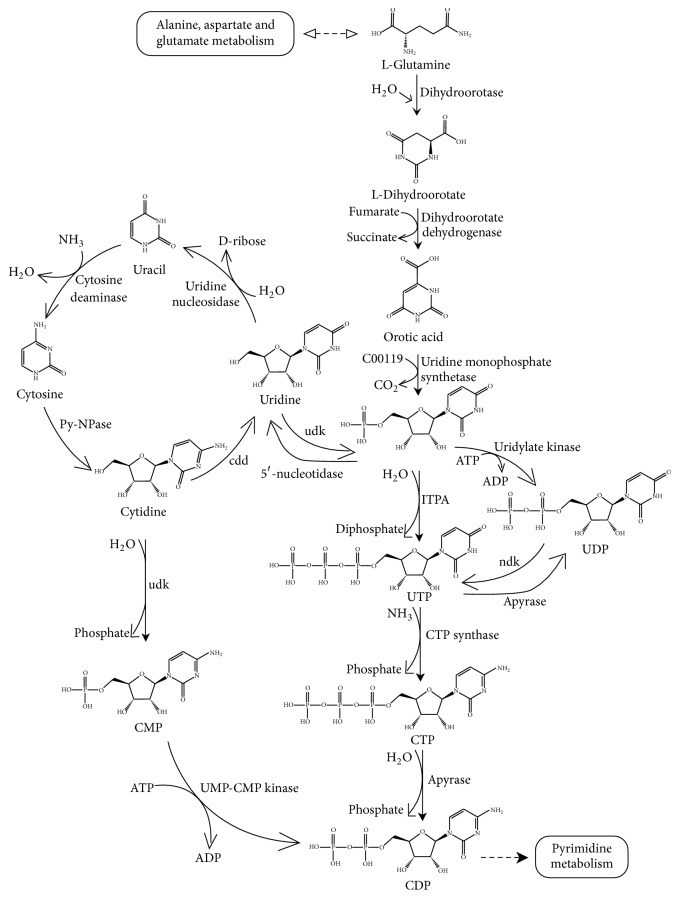
*Biosynthetic pathway of pyrimidine nucleotides in H. sinensis.* The biosynthetic pathway of pyrimidine nucleotides in* H. sinensis* was constructed based on KEGG annotation of the* H. sinensis* transcriptome. Dihydroorotase, dihydroorotate dehydrogenase, uridine monophosphate synthetase, 5′-nucleotidase, uridine nucleosidase, udk, UMP-CMP kinase, ndk, apyrase, ITPA, CTP synthase, and cdd involved in the biosynthetic pathway of pyrimidine nucleotides were annotated in the* H. sinensis* transcriptome.

Based on the annotated pyrimidine metabolic pathway (Figure S2, Supporting Information), the biosynthetic pathway of pyrimidine nucleosides in* H. sinensis*, which started from glutamine, was constructed (Figure 2). In this biosynthetic pathway, dihydroorotase that converts glutamine to dihydroorotate, dihydroorotate dehydrogenase that converts dihydroorotate to orotic acid, uridine monophosphate synthetase that converts orotic acid to UMP, 5′-nucleotidase that converts UMP to uridine, uridine nucleosidase that converts uridine to uracil, uridine kinase (udk) that converts cytidine to CMP, uridine/cytidine monophosphate (UMP-CMP) kinase that converts CMP to CDP, nucleoside-diphosphate kinase (ndk) that converts UDP to UTP, apyrase that converts CTP to CDP, inosine triphosphate pyrophosphatase (ITPA) that converts UMP to UTP, CTP synthase that converts UTP to CTP, and cytidine deaminase (cdd) that converts cytidine to uridine were annotated in the* H. sinensis* transcriptome. Specifically, Unigene8547_All, Unigene8750_All, Unigene11749_All, and Unigene8448_All were annotated to* dihydroorotase*, Unigene13687_All and Unigene6100_All were annotated to* dihydroorotate dehydrogenase*, Unigene3048_All, Unigene3106_All, and Unigene3256_All were annotated to* uridine monophosphate synthetase*, Unigene10391_All and Unigene11624_All were annotated to 5′*-nucleotidase*, Unigene17827_All was annotated to* uridine nucleosidase*, Unigene7973_All was annotated to* uridine kinase*, Unigene11229_All and Unigene11252_All were annotated to* UMP-CMP kinase*, Unigene17829_All was annotated to* nucleoside-diphosphate kinase* (ndk), Unigene1156_All was annotated to* apyrase*, Unigene5342_All was annotated to ITPA, Unigene6007_All was annotated to CTP synthase, and, finally, Unigene11624_All was annotated to* cytidine deaminase* (cdd).

### 3.2. Verifying Biosynthetic Pathways of Purine and Pyrimidine Nucleosides

**Figure 3:**
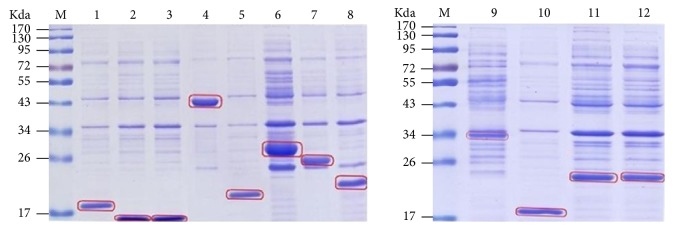
*SDS-PAGE of expression products of biosynthetic genes of purine nucleotides in H. sinensis.* Lane M, the protein marker, lane 1,* purA*, lane 2,* purB*, lane 3,* purC*, lane 4,* purD2*, lane 5,* purE1*, lane 6,* purE2*, lane 7,* purF2*, lane 8,* purF3*, lane 9,* purG2*, lane 10,* purG1*, lane 11,* purD1*, and lane 12,* purF1*.

**Figure 4:**
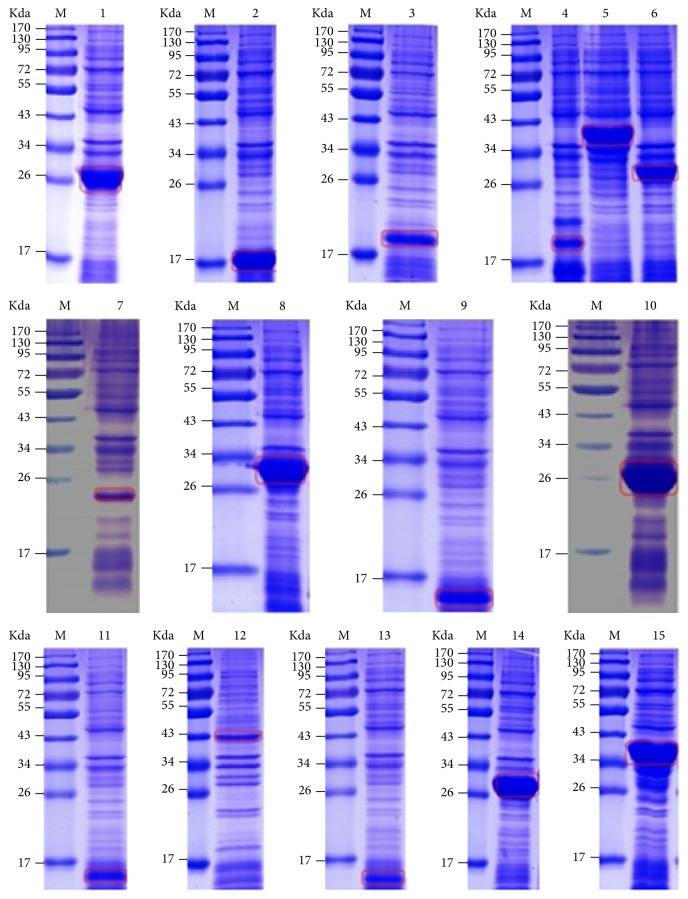
*SDS-PAGE of expression products of biosynthetic genes of pyrimidine nucleotides in H. sinensis.* Lane 1,* pyrA3*, lane 2,* pyrB1*, lane 3,* pyrB2*, lane 4,* pyrC1*, lane 5,* pyrC2*, lane 6,* pyrC3*, lane 7,* pyrD1*, lane 8,* pyrE*, lane 9,* pyrF*, lane 10,* pyrG2*, lane 11,* pyrH*, lane 12,* pyrI*, lane 13,* pyrJ*, lane 14,* pyrK*, and lane 15,* pyrL*.

The genes involved in the biosynthetic pathways of purine and pyrimidine nucleotides were verified by PCR. One* punA* gene, one adenosine kinase gene, one* APRT* gene, two* AMP deaminase* genes, two* IMP dehydrogenase* genes, three* GMP synthase* genes, and two* guanine deaminase* genes were successfully cloned (Figure S3, Supporting Information), and the corresponding proteins expressed in* E. coli* BL21 were detected by SDS-PAGE (Figure 3). In addition, four* dihydroorotase* genes, two* dihydroorotate dehydrogenase* genes, three* uridine monophosphate synthetase* genes, two 5′*-nucleotidase *genes, one* uridine nucleosidase* gene, one* udk* gene, two* UMP-CMP kinase* genes, one* ndk* gene, one* apyrase* gene, one* ITPA* gene, one* CTP synthase* gene, and one* cdd* gene were successfully cloned (Figure S4, Supporting Information). The corresponding proteins expressed in* E. coli* BL21 were detected by SDS-PAGE (Figure 4). Moreover, the gene and protein sequences of these enzymes have been deposited in GenBank (http://www.ncbi.nlm.nih.gov/WebSub/?tool=genbank), and the corresponding GenBank accession numbers are shown in Tables S5 and S6. These results indicated that the enzymes involved in biosynthetic pathways of purine and pyrimidine nucleosides could be produced or detected in* H. sinensis*.

### 3.3. The Differential Expression Analysis of Biosynthetic Genes

**Figure 5:**
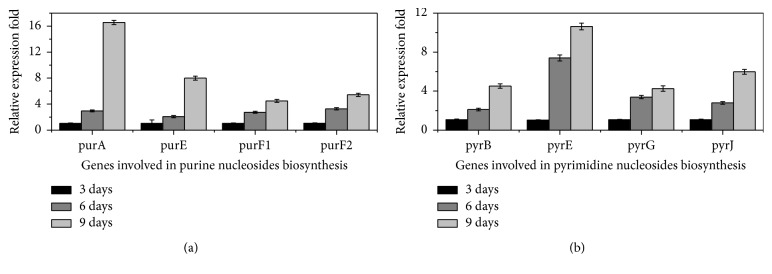
*Significantly upregulated genes involved in biosynthetic pathways of purine and pyrimidine nucleotides in H. sinensis.* (a) Upregulated genes involved in purine nucleotide biosynthesis. (b) Upregulated genes involved in pyrimidine nucleotide biosynthesis.

To detect the differential expression genes (DEGs) involved in the biosynthetic pathways of purine and pyrimidine nucleosides during different culture periods, an efficient procedure of real-time PCR assay with significant specificity and sensitivity was performed. For purine nucleotide biosynthesis, 7 upregulated and 1 downregulated genes were quantified by real-time PCR (Figure S5, Supporting Information). As shown in Figure 5, the relative expression level was upregulated 16.56-fold for* purA* gene, 8-fold for* purE* gene, 4.5-fold for* purF1* gene, and 5.43-fold for* purF2* gene, when 9-day samples were compared with 3-day samples. For pyrimidine nucleoside biosynthesis, 10 upregulated and 3 downregulated genes were quantified by real-time PCR (Figure S6, Supporting Information). Meanwhile, the relative expression level of* pynB* gene was upregulated 4.54-fold,* pynE* gene was upregulated 10.63-fold,* pynG* gene was upregulated 4.26-fold, and* pynJ* gene was upregulated 5.98-fold, when 9-day samples were compared with 3-day samples (Figure 5). These results indicated that* punA*,* IMP dehydrogenase,* and* GMP synthase* genes were overexpressed and played significant or stimulative roles in purine nucleotide biosynthesis.* Dihydroorotate dehydrogenase*,* uridine nucleosidase*,* UMP-CMP kinase,* and* ITPA* genes also played key roles in pyrimidine nucleoside biosynthesis.

### 3.4. Assay of Intracellular Nucleoside Production

**Figure 6:**
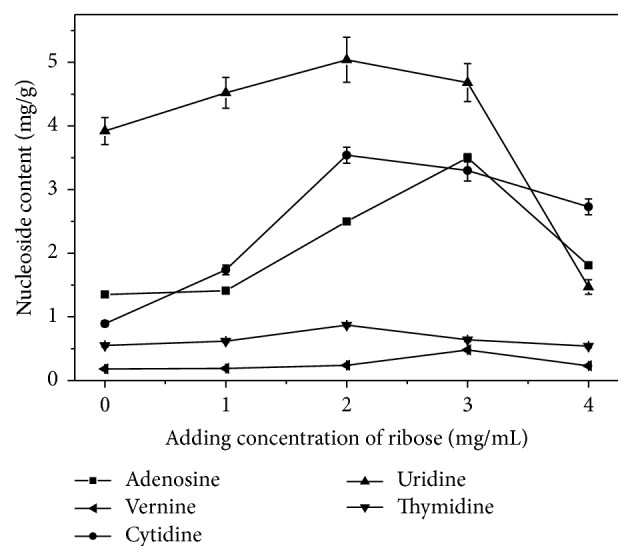
The effects of adding different concentrations of ribose on nucleotide production in* H. sinensis.*

**Figure 7:**
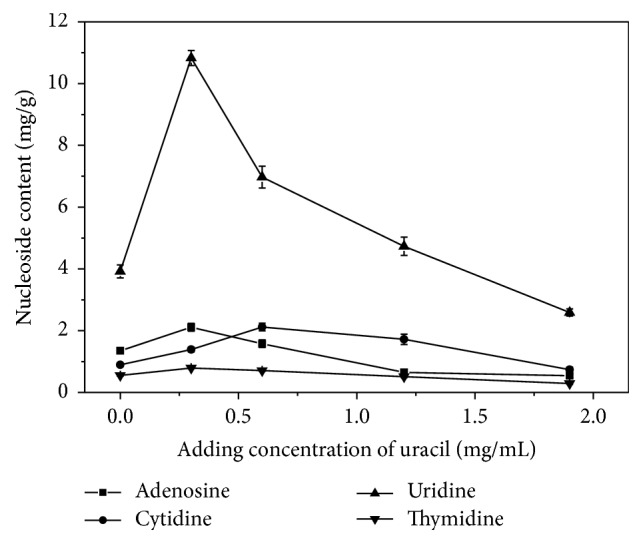
The effects of adding different concentrations of uracil on nucleotide production in* H. sinensis.*

The effects of different additives on nucleoside production were investigated by fermentation regulation in the submerged fermentation of* H. sinensis*. The results indicated that the outputs of adenosine, vernine, cytidine, uridine, and thymidine produced by* H. sinensis* were obviously enhanced compared with the control. For uridine production, the uridine content reached 5.04 mg/g with 28.6% increase over the control when the added concentration of ribose was 2 mg/mL (Figure 6), which was lower than the maximum content of uridine with 10.83 mg/g when the added concentration of uracil was 0.3 mg/mL (Figure 7), indicating that adding uracil was much better than ribose for uridine production. For adenosine production, the maximum content of adenosine reached 3.5 mg/g when the added concentration of ribose was 3 mg/mL (Figure 6), which was a 159.3% increase over the control and better than adenosine content with 2.11 mg/g when the added concentration of uracil was 0.3 mg/mL (Figure 7), indicating that adding ribose was better than uracil for adenosine production. For cytidine production, the maximum content of cytidine reached 3.54 mg/g when the added concentration of ribose was 2 mg/mL (Figure 6), which was a 296% increase over the control and better than the cytidine content of 2.12 mg/g when the added concentration of uracil was 0.6 mg/mL (Figure 7), indicating that adding ribose was better than uracil for cytidine production. In addition, for vernine and thymidine production, the maximum content of vernine reached 0.48 mg/g when the added concentration of ribose was 3 mg/mL (Figure 6), which was a 166.7% increase over the control, and the maximum content of thymidine reached 0.87 mg/g when the added concentration of ribose was 2 mg/mL (Figure 6), indicating that the addition of ribose was more effective for the accumulation of thymidine compared with the addition of uracil.

## 4. Discussion

*O. sinensis* is well known to have been regarded as a traditional medicine or healthful food for a long time in China [26]. To meet the requirements of the market, submerged cultivation of* H. sinensis* provided an environmentally friendly way to resolve the demand [27, 28]. In this study, nucleoside production in* H. sinensis* by submerged fermentation, especially after fermentation regulation, was satisfying or better compared with the nucleoside production reported in previous studies [6, 7], indicating that the submerged fermentation of* H. sinensis* had obvious advantages over culture of wild* O. sinensis*. Furthermore, most of the current studies focused on its medical applications, and there was little genetic information for* O. sinensis* [3]. The availability of transcriptome sequencing could lead to the identification and manipulation of candidate genes for the biosynthetic pathways of bioactive compounds.

As one of important bioactive compounds in* H. sinensis*, nucleoside content is a key factor controlling the quality of products in the submerged fermentation of* H. sinensis*. To date, there are few reports about investigating nucleoside production by submerged fermentation of* H. sinensis*, and the regulating biosynthetic pathways of the nucleosides in* H. sinensis* seem significant and important for enhancing the nucleoside production. Unfortunately, the biosynthetic pathway of the nucleosides has not been determined in* H. sinensis*. In plants, nucleotides could be synthesized from 5-phosphoribosyl-1-pyrophosphate and simple molecules or could be derived from preformed nucleosides and nucleobases via salvage reactions. Nucleotides are degraded to simple metabolites, and this process permits the recycling of phosphate, nitrogen, and carbon into central metabolic pools [29]. The biosynthesis of 3′-deoxyadenosine (cordycepin) in* Cordyceps militaris* has been investigated using [U-^14^C]adenosine and [3-^3^H]ribose, with the ^14^C ratio in the adenine: ribose of the [U-^14^C]adenosine added to the 3′-deoxyadenosine producing cultures of* C. militaris*, which suggested that the formation of 2′-deoxynucleotide and 3′-deoxyadenosine may share a similar synthetic mechanism [30]. Zheng et al. carried out genome sequencing of* C. militaris* and constructed biosynthetic pathways for purine and adenosine based on the KEGG annotation, and it is found that the 5′-nucleotidase was an important enzyme involved in purine and adenosine biosynthesis [31]. The transcriptome of the medicinal* O. sinensis* fruiting body was analyzed by RNA_Seq; a proposed biosynthetic pathway for 3′-deoxyadenosine was constructed, which suggested that 5′-nucleotidase was an important enzyme involved in producing 3′-deoxyadenosine from 3′-dAMP. Adenosine kinase, adenylate kinase, and 5′-nucleotidase, which are involved in adenosine metabolic pathway and show the effects of phosphorylation and dephosphorylation, may also be involved in the biosynthetic pathway of 3′-deoxyadenosine [32]. These previous studies provided evidences and references for the constructions of the biosynthetic pathways of nucleosides in* H. sinensis*, and the putative biosynthetic pathway had positive effects in guiding the enhancement of nucleoside production.

Differential expression analysis was frequently conducted to screen key genes in biosynthetic pathway, and real-time PCR has commonly been applied in relative expression level analysis for decades [33–35]. Gene annotation and differential expression analysis by real-time PCR identified 464 transcripts that may be involved in phytohormone catabolism and biosynthesis, hormone signal, seed dormancy, seed maturation, cell wall growth, and circadian rhythms, particularly. Relative expression levels analysis showed that eleven phytohormone-related genes and five other genes have different expression patterns in the embryo and endosperm in the seed stratification process of* Paris polyphylla* [33]. Two proteases that are known to be directly involved in the process of pathogenesis in the entomopathogenic fungi* Beauveria bassiana* were identified through a comparative analysis of gene expression patterns and then verified by real-time PCR [34]. The differential expression of thirteen PHB accumulation-related genes was investigated by real-time PCR, and thirteen genes showed upregulated when the initial C/N ratio was 2.4, indicating that these genes played the important roles in PHB metabolism in* Acidiphilium cryptum* [35]. These previous studies suggested that real-time PCR is a reliable way to identify key genes involved in biosynthetic pathway. To our knowledge, there are few reports about differential expression analysis in* H. sinensis*, and no information about the regulation of biosynthetic pathways for production of secondary metabolites from* H. sinensis*. In this current study, significantly upregulated genes involved in the biosynthetic pathways of purine and pyrimidine nucleosides were identified by real-time PCR. Based on the results of the differential expression analysis, the uridine nucleosidase gene was significantly upregulated and uridine nucleosidase was considered as a key enzyme involved in the biosynthetic pathway of pyrimidine nucleosides. This study yielded more insight into the biosynthetic process for secondary metabolites and opened a significant way to determine key enzymes involved in a biosynthetic pathway.

Fermentation regulation was reported to enhance the production of target products with satisfying effects [20]. In this study, after fermentation regulation of* H. sinensis* based on biosynthetic pathway and differential expression analysis, the uridine production reached 5.04 mg/g with an increase of 28.6% when 2 mg/mL ribose was added, while uridine production reached 10.83 mg/g with a rise of 176.3% when 0.3 mg/mL uracil was added, indicating that the product inhibition for uridine nucleosidase by uracil was stronger than that by ribose. Some previous studies showed that ribose is an important precursor of nucleosides and uracil is usually considered as an intermediate in nucleoside biosynthesis [30, 36]. In this study, adenosine, cytidine, vernine, and thymidine productions by adding ribose were all better than those by adding uracil, which indicated that ribose played more important role than uracil in nucleoside biosynthesis. This study suggested that the effective fermentation regulation could be applied to improve the production of bioactive compounds and also demonstrated the availability of the constructed biosynthetic pathways.

## 5. Conclusion

In this study, biosynthetic pathways of purine and pyrimidine nucleosides were constructed and verified. Key genes were determined by real-time PCR, and* punA*,* IMP dehydrogenase,* and* GMP synthase* genes involved in purine nucleotide biosynthesis were found upregulated 16.56-fold, 8-fold, and 5.43-fold, respectively. In addition,* dihydroorotate dehydrogenase*,* uridine nucleosidase*,* UMP-CMP kinase,* and* ITPA* genes involved in pyrimidine nucleoside biosynthesis were found upregulated 4.53-fold, 10.63-fold, 4.26-fold, and 5.98-fold, respectively. Regulation of* H. sinensis* fermentation was further conducted based on the biosynthetic pathways and gene differential expressions. Uridine, adenosine, and cytidine production, respectively, yielded an increase of 28.6%, 159.3%, and 296% when ribose was added. In addition, uridine, adenosine, and cytidine production, respectively, had an increase of 176.3%, 56.3%, and 137.1% when uracil was added.

## Supplementary Materials

Supplementary Materials Table S1. The primers used for cloning and expressing genes involved in biosynthetic pathway of purine nucleosides. Table S2. The primers used for cloning and expressing genes involved in biosynthetic pathway of pyrimidine nucleosides. Table S3. The primers involved in purine nucleosides biosynthesis used for real-time PCR. Table S4. The primers involved in pyrimidine nucleosides biosynthesis used for real-time PCR. Table S5. ORF analysis of functional genes involved in biosynthetic pathway of purine nucleotides in* H. sinensis*. Table S6. ORF analysis of functional genes involved in biosynthetic pathway of pyrimidine nucleotides in* H. sinensis*. Figure S1. The annotated purine metabolism pathway in* H. sinensis*. The red marks which stand for the corresponding enzymes were annotated in* H. sinensis* annotation dataset. Figure S2. The annotated pyrimidine metabolism pathway in* H. sinensis*. The red marks which stand for the corresponding enzymes were annotated in* H. sinensis* annotation dataset. Figure S3. Agarose gel electrophoresis of PCR products in biosynthetic pathway of purine nucleotides. Line A stands for purA, lane B1 stands for purB, lane C stands for purC, lane D1 stands for purD1, lane D2 stands for purD2, lane E1 stands for purE1, lane E2 stands for purE2, lane F1 stands for purF1, lane F2 stands for purF2, lane F3 stands for purF3, lane G1 stands for purG1, and lane G2 stands for purG2. Figure S4. Agarose gel electrophoresis of PCR products in biosynthetic pathway of pyrimidine nucleotides. Lane A1 stands for pyrA1, lane A2 stands for pyrA2, lane A3 stands for pyrA3, lane A4 stands for pyrA4, lane B1 stands for pyrB1, lane B2 stands for pyrB2, lane C1 stands for pyrC1, lane C2 stands for pyrC2, lane C3 stands for pyrC3, lane D1 stands for pyrD1, lane D2 stands for pyrD2, lane E stands for pyrE, lane F stands for pyrF, lane G1 stands for pyrG1, lane G2 stands for pyrG2, lane H stands for pyrH, lane I stands for pyrI, lane J stands for pyrJ, lane K stands for pyrK, and lane L stands for pyrL. Figure S5. Differential expression analysis of other four functional genes involved in biosynthetic pathway of purine nucleotides. Figure S6. Differential expression analysis of other nine functional genes involved in biosynthetic pathway of pyrimidine nucleosides.
