# Supplementary material for: Enhancement of Nucleoside Production in Hirsutella sinensis Based on Biosynthetic Pathway Analysis
Source: Biomed Res Int. 2017 Nov 29;2017:2520347. doi: 10.1155/2017/2520347 (PMC5733210; doi:10.1155/2017/2520347)

Supporting Information

Enhancement of nucleoside production in *Hirsutella sinensis* based on biosynthetic pathway analysis

Zhi-Qiang Liu[a], Bo Zhang[a], Shan Lin[a], Peter James Baker[a], Mao-Sheng Chen[b], Ya-Ping Xue[a], Hui Wu[c], Feng Xu[c], Shui-Jin Yuan[c], Yi Teng[c], Ling-Fang Wu[c], Yu-Guo Zheng[a]*

[1] Key Laboratory of Bioorganic Synthesis of Zhejiang Province, College of Biotechnology and Bioengineering, Zhejiang University of Technology, Hangzhou 310014, China

[2] Department of Nepharology, Zhejiang Provincial People's Hospital, Hangzhou 310014, China

[3] East China Pharmaceutical Group Limited Co., Ltd, Hangzhou 311000, China

* **Corresponding author**: Yu-Guo Zheng, Key Laboratory of Bioorganic Synthesis of Zhejiang Province, College of Biotechnology and Bioengineering, Zhejiang University of Technology, Hangzhou 310014, P. R. China; zhengyg@zjut.edu.cn; Tel: +86-571-88320614; Fax: +86-571-88320630.

Table S1. The primers used for cloning and expressing genes involved in biosynthetic pathway of purine nucleosides

Genes	Primers	Sequence (5’-3’)
purA	purA-F	ATTGAATTCATGACCATGCCAGACTCGTC
	purA-R	AGTAAGCTTCTAACGCGTGCCGTTAGAG
purB	purB-F	ATTGAATTCATGCTGTCTCACTCGCGGTTAG
	purB-R	ATTAAGCTTCTAACATTCGGCGCACGCG
purC	purC-F	ATTGAATTCATGCTGCCGGCGGCCG
	purC-R	GCCAAGCTTCTACTCGAGTGGCAGATTGACC
purD1	purD1-F	ATTGAGCTCATGGTGAAGACGAACGTGC
	purD1-R	ATTAAGCTTCTAGTTCCGTACCCTGCCAC
purD2	purD2-F	ATTGAATTCATGCTGCTGGCCAAGCTGG
	purD2-R	ATTAAGCTTCTAGCCGGCCTCGAAGAG
purE1	purE1-F	ATTGAATTCATGCTGCATCCGCAATGGC
	purE1-R	ATTAAGCTTCTACGAGGATGAAGCCGTTTTC
purE2	purE2-F	ATTGAATTCATGGTGACCGACCTCACC
	purE2-R	GCCGAGCTCTCATGCATACAGCTTCTTCTC
purF1	purF1-F	GTTGAATTCATGCTGATCTCCCATCACTCC
	purF1-R	ATTAAGCTTCTACATCACAACGTAGGCGGG
purF2	purF2-F	ATTGAATTCATGCTGCTCCAGAACTTTGCCG
	purF2-R	ATTAAGCTTCTAAGCCGGCAGCCCGCC
purF3	purF3-F	ATTGAATTCATGCTGACTGCCAACCACCTC
	purF3-R	GCCAAGCTTCTAGACGAGCTTGTCAAAGTG
purG1	purG1-F	ATTGAATTCATGAAGCCCGGGAAGAAAAAC
	purG1-R	ATTAAGCTTCTAGGTCTCGGGGACGCG
purG2	purG2-F	GCCGAATTCATGCTGCAAAAGTACACGTTC
	purG2-R	ATTAAGCTTTTACGGGGGGCCCGCGG

Table S2. The primers used for cloning and expressing genes involved in biosynthetic pathway of pyrimidine nucleosides

Genes	Primers	Sequence (5’-3’)
pyrA1	pyrA1-F	AGTGGATCCATGACGCCTAATATGCTGGCTG
	pyrA1-R	ACCAAGCTTTCAGGCCCTGATGATTTGCATG
pyrA2	pyrA2-F	AGTGAATTCATGGCAGTCAAGCGCGACTAC
	pyrA2-R	AGTAAGCTTTTACAGCGGCTTGCCGCCG
pyrA3	pyrA3-F	ATTGAATTCATGACCGGCCGGTTCGTCCTC
	pyrA3-R	ATAAAGCTTTCAGGTGAGGTCCAGGGCGGGG
pyrA4	pyrA4-F	AGCGAATTCATGCAGCTCAGATCTATCCAAC
	pyrA4-R	ATTGAGCTCTTACGCAGCCTCCTCCCACC
pyrB1	pyrB1-F	AGCGAATTCATGGCCATGATGGAGCAAGG
	pyrB1-R	AGCAAGCTTTCAGTTCCGCTGTCTCATCTC
pyrB2	pyrB2-F	ATAGAATTCATGTCGGCTTCAACGGCAGC
	pyrB2-R	ATTAAGCTTTCAGTCCCAGCGATCGCGCG
pyrC1	pyrC1-F	AGAGAATTCATGTTGGCTGACGCTAGGGTC
	pyrC1-R	ATAAAGCTTTCACCCTCTGCCGCACCAACTTG
pyrC2	pyrC2-F	ATTGGATCCATGCAGTACACTGGCGGCTC
	pyrC2-R	ATTGAGCTCTCAGCGAACTCTCTCGAGGTATG
pyrC3	pyrC3-F	ATAGAATTCATGGCGTCTCAGCTGCCCGCG
	pyrC3-R	AGCAAGCTTTCACTTAGTTGGCGGGTACTTG
pyrD1	pyrD1-F	ATAGAATTCATGGAAACAGCTCCCCTCGGC
	pyrD1-R	AGCAAGCTTTCACGAAAGCCGCTCTACTATC
pyrD2	pyrD2-F	ATAGAATTCATGGGCAACGACACGACGGCG
	pyrD2-R	AGGAAGCTTTCACGTTGCCCGCATCTTGATTC
pyrE	pyrE-F	AGCGAATTCATGATGACCAGCACGCTAAAAAT
	pyrE-R	ATTAAGCTTTCAAAGGAGCCCTCGGTGACG
pyrF	pyrF-F	AGCGAATTCATGGATATTGAGACACCCTG
	pyrF-R	ATTAAGCTTTCACCCGTGGTCGACAAGCACC
pyrG1	pyrG1-F	ACTGAATTCATGCCGTGTCGCTCATCTGC
	pyrG1-R	AGGGTCGACTTATGAGTTTCTACGGTGTTC
pyrG2	pyrG2-F	ATAGAATTCATGGCCTCTCGCCTGGCCCCC
	pyrG2-R	ATCAAGCTTTCAAAAGGAGGTGCCGAGCTTC
pyrH	pyrH-F	ATAGAATTCATGTGCTCCTTGGAGGGGTGG
	pyrH-R	AGCAAGCTTTCACTCCCAATTCAAACTCGC
pyrI	pyrI-F	AGCGAATTCATGGGTAAGAACTCGCAATATG
	pyrI-R	ATAAAGCTTTCACAAATCCTCACGCGGCGTC
pyrJ	pyrJ-F	ATAGAATTCATGGACCCCTGCGTCACCTTTG
	pyrJ-R	AGTAAGCTTTCAAAGATCGGCGGCTTTGTGGC
pyrK	pyrK-F	ATAGAATTCATGCGCTGCGGTCGGAAACTG
	pyrK-R	ACGAAGCTTCTAAAAGCAAGAATTATCAGG
pyrL	pyrL-F	ATAGAATTCATGGGCAACGACACGACGGCG
	pyrL-R	AGGAAGCTTTCACGTTGCCCGCATCTTGATTC

Table S3. The primers involved in purine nucleosides biosynthesis used for real-time PCR

Genes	Primers	Sequence (5'-3')
18S rRNA	18S-F	GCAGTGGCATCTCTCAGTC
	18S-R	TCATCGATGCCAGAACC
purA	PurA-F	ACCCCCGGATCAAGCTACTAG
	PurA-R	GTTGCGCGTCGTGTGTTC
purB	PurB-F	CTGTCTCACTCGCGGTTAGGT
	PurB-R	CCCAATGGACCACGACAAGT
purC	PurC-F	CACCCGTGGCAATGATGTC
	PurC-R	GGAGTACGGCAAAGACCTCTTC
purD	PurD-F	CCGGACAGAAGGGAAGAGTTT
	PurD-R	GTCACGTAGCGTTTGAGAACGT
purE	PurE-F	TGATCTCCCGGTCCGACTT
	PurE-R	TGCTTGCTGTCGGGTAGCT
purF1	PurF1-F	GCAAAAACATCTCGGCATCA
	PurF1-R	CGGTCAAGGAACAGCTTGGA
purF2	PurF2-F	AAATGGCCCCCGTAAACG
	PurF2-R	GATGGTGTCGAAAGTGCTGTGA
purG	PurG-F	CGTGTTTGGGTGGGAGACTT
	PurG-R	ACCTTTTTGGTGTTTCGATCGT

Table S4. The primers involved in pyrimidine nucleosides biosynthesis used for real-time PCR

Genes	Primers	Sequence (5’-3’)
pyrA	PyrA-F	GGCGTGCCCCTTTTCAT
	PyrA-R	GCAGCTTCTCGCTCATGCA
pyrB	PyrB-F	CATGTCCATGATGAAGCTCCTGTA
	PyrB-R	AGGTGAGCGCCAAAAACG
pyrC1	PyrC1-F	CGTCGCTTGCCAATGCT
	PyrC1-R	GACGTCTTGACCTCGTAGGGATA
pyrC2	PyrC2-F	TCGTGGCAGGCATTGTTG
	PyrC2-R	GTCTGCAGCAGGGAGCTTCT
pyrD	PyrD-F	GCGACACCACGGACAAGAT
	PyrD-R	GCCCAGCGGTTCCAAAA
pyrE	PyrE-F	TGATGACCAGCACGCTAAAAA
	PyrE-R	TCCACAACGCCTCGACTCA
pyrF	PyrF-F	CTACTCGCCGCATGTGCTTA
	PyrF-R	TTCCGATGTCAAGGAGCTCAA
pyrG	PyrG-F	TACGGCGCGAACAAGCA
	PyrG-R	CAAAGCTCTGCGTGATGAAGTCT
pyrH	PyrH-F	CGCCCAGAAGGAGATCAAAC
	PyrH-R	TGCCGGAGGAACGGTAGTT
pyrI	PyrI-F	TAGGCATTCCCCGAGTCATC
	PyrI-R	CTTGGCATTTCCCACTGCTT
pyrJ	PyrJ-F	ACCCCTGCGTCACCTTTGT
	PyrJ-R	GTGCTCTGAATCCGGATGGT
pyrK	PyrK-F	GGCCACATCGGAGGAGTTT
	PyrK-R	CCTTCAGGCATGAAAATGATAAGG
pyrL	PyrL-F	CGCGGATCCGAATTCG
	PyrL-R	TGATCCACCCCTCCAAGGA

Table S5. ORF analysis of functional genes involved in biosynthetic pathway of purine nucleotides in *H. sinensis*

Enzymes	Location of genes	ORFs	Length (bp)	GenBank accession numbers
punA	Unigene14697_All	purA	855	GCQL01014666
Adenosine kinase	Unigene1297_All	purB	326	GCQL01001297
APRT	Unigene12674_All	purC	371	GCQL01012671
AMP deaminase	Unigene13557_All	purD1	378	GCQL01013554
	Unigene3652_All	purD2	1032	GCQL01003651
IMP dehydrogenase	Unigene6288_All	purE1	407	GCQL01006286
	Unigene9455_All	purE2	1047	GCQL01009452
GMP synthase	Unigene11031_All	purF1	331	GCQL01011028
	Unigene11950_All	purF2	553	GCQL01011947
	Unigene13696_All	purF3	490	GCQL01013693
Guanine deaminase	Unigene7330_All	purG1	328	GCQL01007327
	Unigene9274_All	purG2	753	GCQL01009271

Table S6. ORF analysis of functional genes involved in biosynthetic pathway of pyrimidine nucleotides in *H. sinensis*

Enzymes	Location of genes	ORFs	Length (bp)	GenBank accession numbers
Dihydroorotase	Unigene8547_All	pyrA1	2718	GCQL01008544
	Unigene8750_All	pyrA2	624	GCQL01008747
	Unigene11749_All	pyrA3	984	GCQL01011746
	Unigene8448_All	pyrA4	1095	GCQL01008445
Dihydroorotate	Unigene13687_All	pyrB1	303	GCQL01013684
dehydrogenase	Unigene6100_All	pyrB2	660	GCQL01006098
Uridine monophosphate	Unigene3048_All	pyrC1	414	GCQL01003047
synthetase	Unigene3106_All	pyrC2	768	GCQL01003105
	Unigene3256_All	pyrC3	738	GCQL01003255
5'-nucleotidase	Unigene10391_All	pyrD1	519	GCQL01010388
	Unigene11624_All	pyrD2	1971	GCQL01011621
Uridine nucleosidase	Unigene17827_All	pyrE	1281	GCQL01017603
udk	Unigene7973_All	pyrF	300	GCQL01007970
UMP-CMP kinase	Unigene11229_All	pyrG1	1266	GCQL01011226
	Unigene11252_All	pyrG2	912	GCQL01011249
ndk	Unigene17829_All	pyrH	216	GCQL01017605
Apyras	Unigene1156_All	pyrI	2049	GCQL01001156
ITPA	Unigene5342_All	pyrJ	168	GCQL01005340
CTP synthase	Unigene6007_All	pyrK	741	GCQL01006005
cdd	Unigene11624_All	pyrL	1971	GCQL01011621

Figure S1. The annotated purine metabolism pathway in *H. sinensis*. The red marks stand for the corresponding enzymes were annotated in *H. sinensis* annotation dataset.

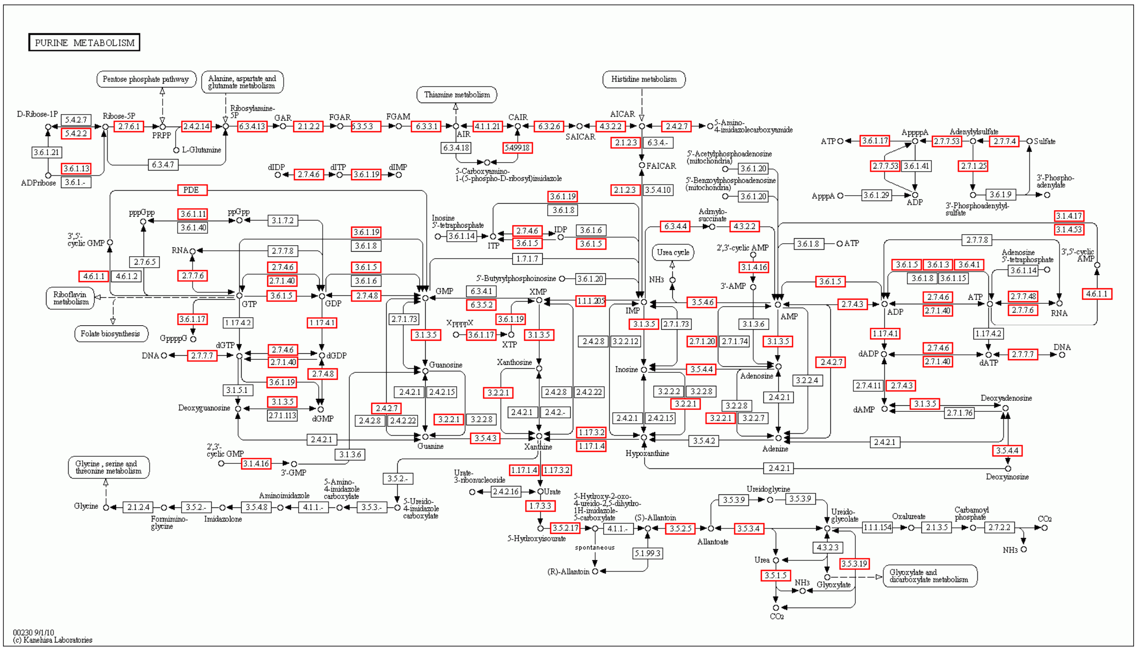

Figure S2. The annotated pyrimidine metabolism pathway in *H. sinensis*. The red marks stand for the corresponding enzymes were annotated in *H. sinensis* annotation dataset.

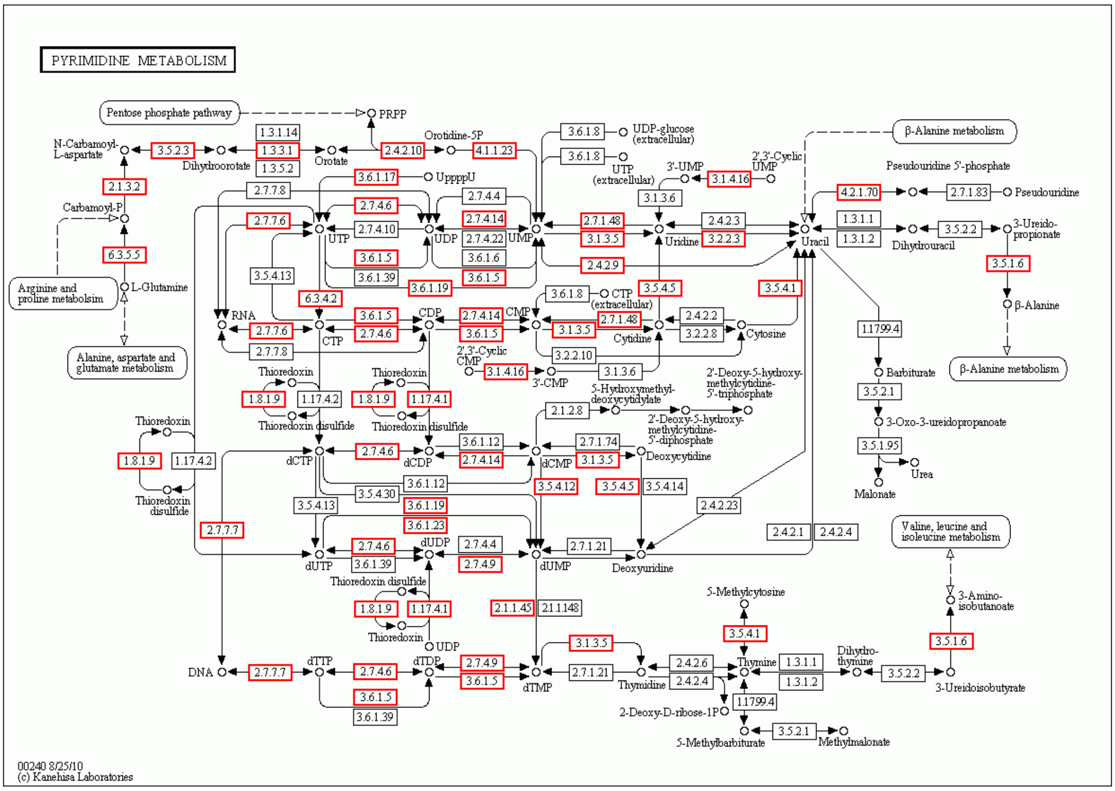

Figure S3. Agarose gel electrophoresis of PCR products in biosynthetic pathway of purine nucleotides. Line A stands for purA, lane B1 stands for purB, lane C stands for purC, lane D1 stands for purD1, lane D2 stands for purD2, lane E1 stands for purE1, lane E2 stands for purE2, lane F1 stands for purF1, lane F2 stands for purF2, lane F3 stands for purF3, lane G1 stands for purG1, and lane G2 stands for purG2.

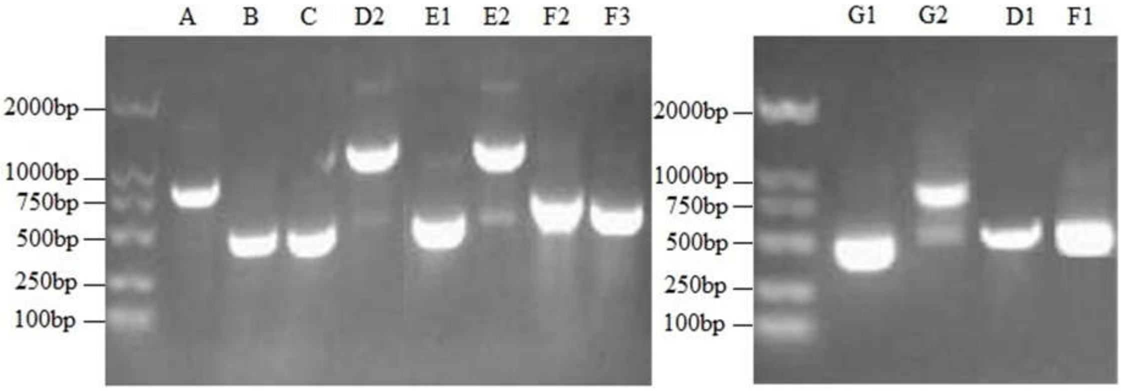

Figure S4. Agarose gel electrophoresis of PCR products in biosynthetic pathway of pyrimidine nucleotides. Lane A1 stands for pyrA1, lane A2 stands for pyrA2, lane A3 stands for pyrA3, lane A4 stands for pyrA4, lane B1 stands for pyrB1, lane B2 stands for pyrB2, lane C1 stands for pyrC1, lane C2 stands for pyrC2, lane C3 stands for pyrC3, lane D1 stands for pyrD1, lane D2 stands for pyrD2, lane E stands for pyrE, lane F stands for pyrF, lane G1 stands for pyrG1, lane G2 stands for pyrG2, lane H stands for pyrH, lane I stands for pyrI, lane J stands for pyrJ, lane K stands for pyrK, and lane L stands for pyrL.

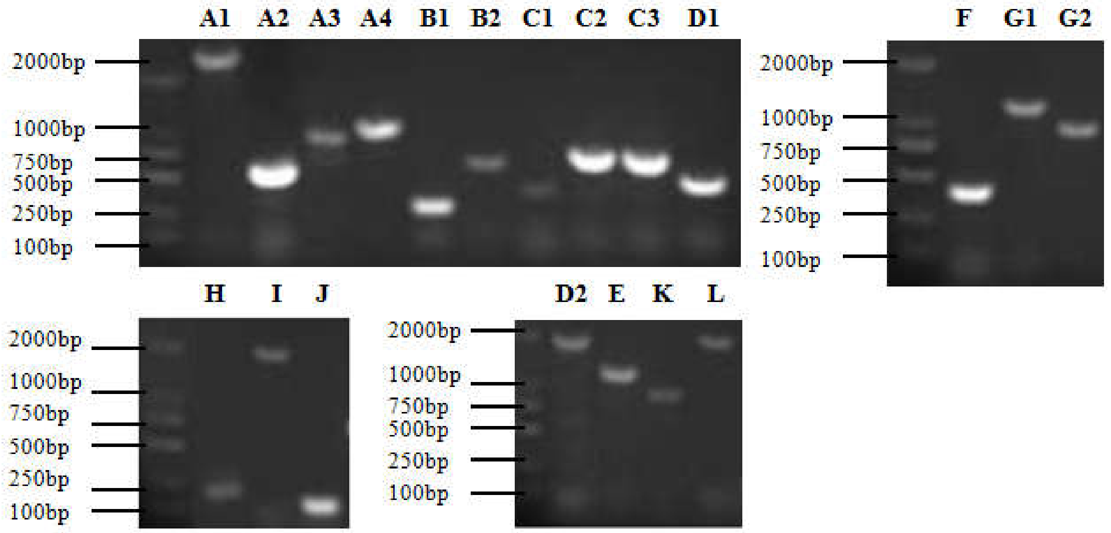

Figure S5. Differential expression analysis of other four functional genes involved in biosynthetic pathway of purine nucleotides.

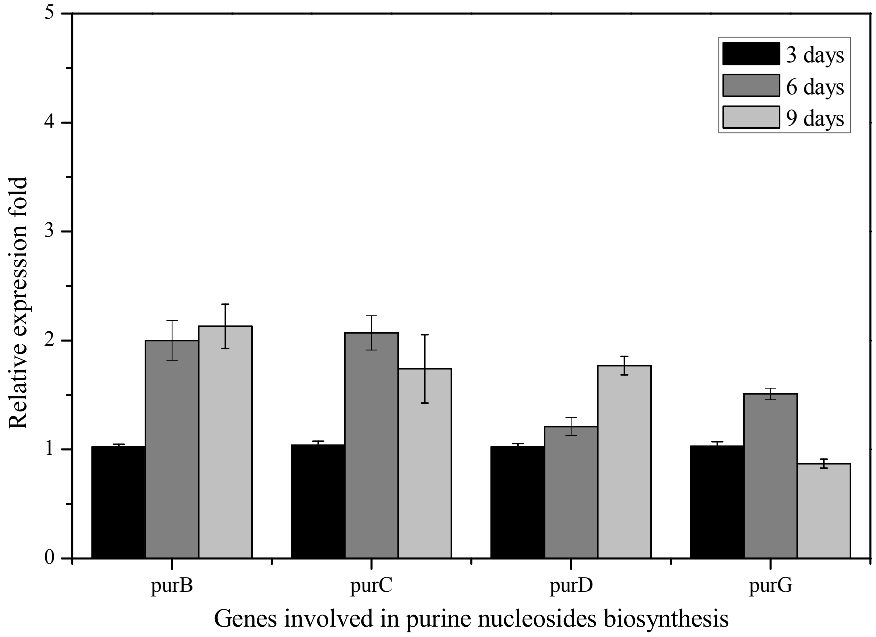

Figure S6. Differential expression analysis of other nine functional genes involved in biosynthetic pathway of pyrimidine nucleosides.

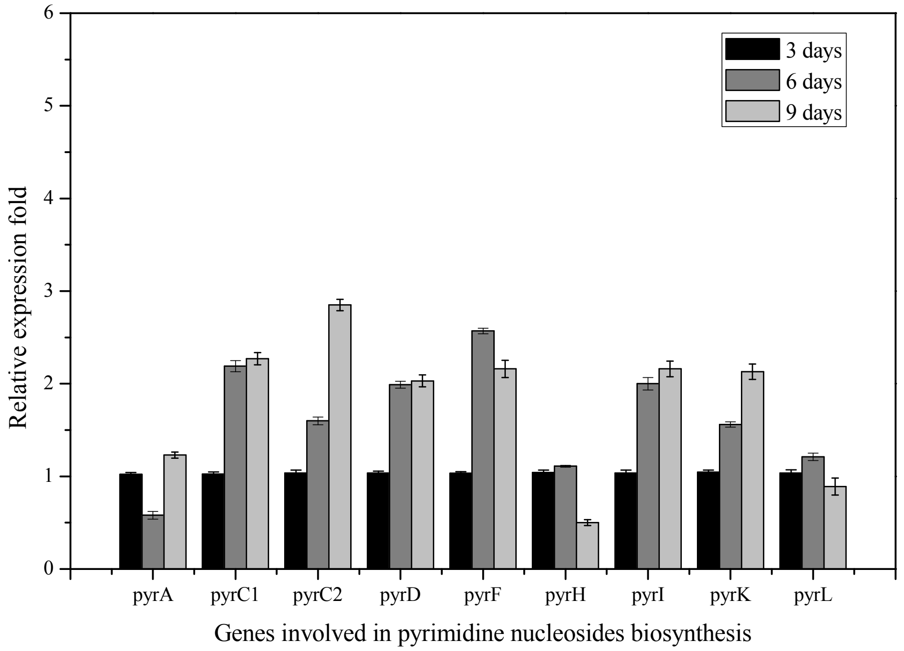

Supplement: Supplementary Materials — Table S1. The primers used for cloning and expressing genes involved in biosynthetic pathway of purine nucleosides. Table S2. The primers used for cloning and expressing genes involved in biosynthetic pathway of pyrimidine nucleosides. Table S3. The primers involved in purine nucleosides biosynthesis used for real-time PCR. Table S4. The primers involved in pyrimidine nucleosides biosynthesis used for real-time PCR. Table S5. ORF analysis of functional genes involved in biosynthetic pathway of purine nucleotides in H. sinensis. Table S6. ORF analysis of functional genes involved in biosynthetic pathway of pyrimidine nucleotides in H. sinensis. Figure S1. The annotated purine metabolism pathway in H. sinensis. The red marks which stand for the corresponding enzymes were annotated in H. sinensis annotation dataset. Figure S2. The annotated pyrimidine metabolism pathway in H. sinensis. The red marks which stand for the corresponding enzymes were annotated in H. sinensis annotation dataset. Figure S3. Agarose gel electrophoresis of PCR products in biosynthetic pathway of purine nucleotides. Line A stands for purA, lane B1 stands for purB, lane C stands for purC, lane D1 stands for purD1, lane D2 stands for purD2, lane E1 stands for purE1, lane E2 stands for purE2, lane F1 stands for purF1, lane F2 stands for purF2, lane F3 stands for purF3, lane G1 stands for purG1, and lane G2 stands for purG2. Figure S4. Agarose gel electrophoresis of PCR products in biosynthetic pathway of pyrimidine nucleotides. Lane A1 stands for pyrA1, lane A2 stands for pyrA2, lane A3 stands for pyrA3, lane A4 stands for pyrA4, lane B1 stands for pyrB1, lane B2 stands for pyrB2, lane C1 stands for pyrC1, lane C2 stands for pyrC2, lane C3 stands for pyrC3, lane D1 stands for pyrD1, lane D2 stands for pyrD2, lane E stands for pyrE, lane F stands for pyrF, lane G1 stands for pyrG1, lane G2 stands for pyrG2, lane H stands for pyrH, lane I stands for pyrI, lane J stands for pyrJ, lane K stands for pyr [file 2520347.f1.zip › revised Supporting Information MS ID 2520347.pdf]
