# Supplementary material for: Enhancement of Nucleoside Production in Hirsutella sinensis Based on Biosynthetic Pathway Analysis
Source: Biomed Res Int. 2017 Nov 29;2017:2520347. doi: 10.1155/2017/2520347 (PMC5733210; doi:10.1155/2017/2520347)
Supplement: Supplementary Materials — Table S1. The primers used for cloning and expressing genes involved in biosynthetic pathway of purine nucleosides. Table S2. The primers used for cloning and expressing genes involved in biosynthetic pathway of pyrimidine nucleosides. Table S3. The primers involved in purine nucleosides biosynthesis used for real-time PCR. Table S4. The primers involved in pyrimidine nucleosides biosynthesis used for real-time PCR. Table S5. ORF analysis of functional genes involved in biosynthetic pathway of purine nucleotides in H. sinensis. Table S6. ORF analysis of functional genes involved in biosynthetic pathway of pyrimidine nucleotides in H. sinensis. Figure S1. The annotated purine metabolism pathway in H. sinensis. The red marks which stand for the corresponding enzymes were annotated in H. sinensis annotation dataset. Figure S2. The annotated pyrimidine metabolism pathway in H. sinensis. The red marks which stand for the corresponding enzymes were annotated in H. sinensis annotation dataset. Figure S3. Agarose gel electrophoresis of PCR products in biosynthetic pathway of purine nucleotides. Line A stands for purA, lane B1 stands for purB, lane C stands for purC, lane D1 stands for purD1, lane D2 stands for purD2, lane E1 stands for purE1, lane E2 stands for purE2, lane F1 stands for purF1, lane F2 stands for purF2, lane F3 stands for purF3, lane G1 stands for purG1, and lane G2 stands for purG2. Figure S4. Agarose gel electrophoresis of PCR products in biosynthetic pathway of pyrimidine nucleotides. Lane A1 stands for pyrA1, lane A2 stands for pyrA2, lane A3 stands for pyrA3, lane A4 stands for pyrA4, lane B1 stands for pyrB1, lane B2 stands for pyrB2, lane C1 stands for pyrC1, lane C2 stands for pyrC2, lane C3 stands for pyrC3, lane D1 stands for pyrD1, lane D2 stands for pyrD2, lane E stands for pyrE, lane F stands for pyrF, lane G1 stands for pyrG1, lane G2 stands for pyrG2, lane H stands for pyrH, lane I stands for pyrI, lane J stands for pyrJ, lane K stands for pyr [file 2520347.f1.zip › revised Supporting Information MS ID 2520347.doc]

**Supporting Information**

**Enhancement of nucleoside production in *Hirsutella sinensis* based on biosynthetic pathway analysis**

Zhi-Qiang Liu^a^, Bo Zhang^a^, Shan Lin^a^, Peter James Baker^a^, Mao-Sheng Chen^b^, Ya-Ping Xue^a^, Hui Wu^c^, Feng Xu^c^, Shui-Jin Yuan^c^, Yi Teng^c^, Ling-Fang Wu^c^, Yu-Guo Zheng^a^*****

^1^ Key Laboratory of Bioorganic Synthesis of Zhejiang Province, College of Biotechnology and Bioengineering, Zhejiang University of Technology, Hangzhou 310014, China

^2^ Department of Nepharology, Zhejiang Provincial People’s Hospital, Hangzhou 310014, China

^3^ East China Pharmaceutical Group Limited Co., Ltd, Hangzhou 311000, China

* **Corresponding author**: Yu-Guo Zheng, Key Laboratory of Bioorganic Synthesis of Zhejiang Province, College of Biotechnology and Bioengineering, Zhejiang University of Technology, Hangzhou 310014, P. R. China; [zhengyg@zjut.edu.cn](mailto:zhengyg@zjut.edu.cn); Tel: +86-571-88320614; Fax: +86-571-88320630.

**Table S1.** The primers used for cloning and expressing genes involved in biosynthetic pathway of purine nucleosides

| Genes | Primers | Sequence (5’-3’) |
| --- | --- | --- |
| purA | purA-F | ATTGAATTCATGACCATGCCAGACTCGTC |
|  | purA-R | AGTAAGCTTCTAACGCGTGCCGTTAGAG |
| purB | purB-F | ATTGAATTCATGCTGTCTCACTCGCGGTTAG |
|  | purB-R | ATTAAGCTTCTAACATTCGGCGCACGCG |
| purC | purC-F | ATTGAATTCATGCTGCCGGCGGCCG |
|  | purC-R | GCCAAGCTTCTACTCGAGTGGCAGATTGACC |
| purD1 | purD1-F | ATTGAGCTCATGGTGAAGACGAACGTGC |
|  | purD1-R | ATTAAGCTTCTAGTTCCGTACCCTGCCAC |
| purD2 | purD2-F | ATTGAATTCATGCTGCTGGCCAAGCTGG |
|  | purD2-R | ATTAAGCTTCTAGCCGGCCTCGAAGAG |
| purE1 | purE1-F | ATTGAATTCATGCTGCATCCGCAATGGC |
|  | purE1-R | ATTAAGCTTCTACGAGGATGAAGCCGTTTTC |
| purE2 | purE2-F | ATTGAATTCATGGTGACCGACCTCACC |
|  | purE2-R | GCCGAGCTCTCATGCATACAGCTTCTTCTC |
| purF1 | purF1-F | GTTGAATTCATGCTGATCTCCCATCACTCC |
|  | purF1-R | ATTAAGCTTCTACATCACAACGTAGGCGGG |
| purF2 | purF2-F | ATTGAATTCATGCTGCTCCAGAACTTTGCCG |
|  | purF2-R | ATTAAGCTTCTAAGCCGGCAGCCCGCC |
| purF3 | purF3-F | ATTGAATTCATGCTGACTGCCAACCACCTC |
|  | purF3-R | GCCAAGCTTCTAGACGAGCTTGTCAAAGTG |
| purG1 | purG1-F | ATTGAATTCATGAAGCCCGGGAAGAAAAAC |
|  | purG1-R | ATTAAGCTTCTAGGTCTCGGGGACGCG |
| purG2 | purG2-F | GCCGAATTCATGCTGCAAAAGTACACGTTC |
|  | purG2-R | ATTAAGCTTTTACGGGGGGCCCGCGG |

**Table S2.** The primers used for cloning and expressing genes involved in biosynthetic pathway of pyrimidine nucleosides

| Genes | Primers | Sequence (5’-3’) |
| --- | --- | --- |
| pyrA1 | pyrA1-F | AGTGGATCCATGACGCCTAATATGCTGGCTG |
|  | pyrA1-R | ACCAAGCTTTCAGGCCCTGATGATTTGCATG |
| pyrA2 | pyrA2-F | AGTGAATTCATGGCAGTCAAGCGCGACTAC |
|  | pyrA2-R | AGTAAGCTTTTACAGCGGCTTGCCGCCG |
| pyrA3 | pyrA3-F | ATTGAATTCATGACCGGCCGGTTCGTCCTC |
|  | pyrA3-R | ATAAAGCTTTCAGGTGAGGTCCAGGGCGGGG |
| pyrA4 | pyrA4-F | AGCGAATTCATGCAGCTCAGATCTATCCAAC |
|  | pyrA4-R | ATTGAGCTCTTACGCAGCCTCCTCCCACC |
| pyrB1 | pyrB1-F | AGCGAATTCATGGCCATGATGGAGCAAGG |
|  | pyrB1-R | AGCAAGCTTTCAGTTCCGCTGTCTCATCTC |
| pyrB2 | pyrB2-F | ATAGAATTCATGTCGGCTTCAACGGCAGC |
|  | pyrB2-R | ATTAAGCTTTCAGTCCCAGCGATCGCGCG |
| pyrC1 | pyrC1-F | AGAGAATTCATGTTGGCTGACGCTAGGGTC |
|  | pyrC1-R | ATAAAGCTTTCACCCTCTGCCGCACCAACTTG |
| pyrC2 | pyrC2-F | ATTGGATCCATGCAGTACACTGGCGGCTC |
|  | pyrC2-R | ATTGAGCTCTCAGCGAACTCTCTCGAGGTATG |
| pyrC3 | pyrC3-F | ATAGAATTCATGGCGTCTCAGCTGCCCGCG |
|  | pyrC3-R | AGCAAGCTTTCACTTAGTTGGCGGGTACTTG |
| pyrD1 | pyrD1-F | ATAGAATTCATGGAAACAGCTCCCCTCGGC |
|  | pyrD1-R | AGCAAGCTTTCACGAAAGCCGCTCTACTATC |
| pyrD2 | pyrD2-F | ATAGAATTCATGGGCAACGACACGACGGCG |
|  | pyrD2-R | AGGAAGCTTTCACGTTGCCCGCATCTTGATTC |
| pyrE | pyrE-F | AGCGAATTCATGATGACCAGCACGCTAAAAAT |
|  | pyrE-R | ATTAAGCTTTCAAAGGAGCCCTCGGTGACG |
| pyrF | pyrF-F | AGCGAATTCATGGATATTGAGACACCCTG |
|  | pyrF-R | ATTAAGCTTTCACCCGTGGTCGACAAGCACC |
| pyrG1 | pyrG1-F | ACTGAATTCATGCCGTGTCGCTCATCTGC |
|  | pyrG1-R | AGGGTCGACTTATGAGTTTCTACGGTGTTC |
| pyrG2 | pyrG2-F | ATAGAATTCATGGCCTCTCGCCTGGCCCCC |
|  | pyrG2-R | ATCAAGCTTTCAAAAGGAGGTGCCGAGCTTC |
| pyrH | pyrH-F | ATAGAATTCATGTGCTCCTTGGAGGGGTGG |
|  | pyrH-R | AGCAAGCTTTCACTCCCAATTCAAACTCGC |
| pyrI | pyrI-F | AGCGAATTCATGGGTAAGAACTCGCAATATG |
|  | pyrI-R | ATAAAGCTTTCACAAATCCTCACGCGGCGTC |
| pyrJ | pyrJ-F | ATAGAATTCATGGACCCCTGCGTCACCTTTG |
|  | pyrJ-R | AGTAAGCTTTCAAAGATCGGCGGCTTTGTGGC |
| pyrK | pyrK-F | ATAGAATTCATGCGCTGCGGTCGGAAACTG |
|  | pyrK-R | ACGAAGCTTCTAAAAGCAAGAATTATCAGG |
| pyrL | pyrL-F | ATAGAATTCATGGGCAACGACACGACGGCG |
|  | pyrL-R | AGGAAGCTTTCACGTTGCCCGCATCTTGATTC |

**Table S3.** The primers involved in purine nucleosides biosynthesis used for real-time PCR

| Genes | Primers | Sequence (5’-3’) |
| --- | --- | --- |
| 18S rRNA | 18S-F | GCAGTGGCATCTCTCAGTC |
|  | 18S-R | TCATCGATGCCAGAACC |
| purA | PurA-F | ACCCCCGGATCAAGCTACTAG |
|  | PurA-R | GTTGCGCGTCGTGTGTTC |
| purB | PurB-F | CTGTCTCACTCGCGGTTAGGT |
|  | PurB-R | CCCAATGGACCACGACAAGT |
| purC | PurC-F | CACCCGTGGCAATGATGTC |
|  | PurC-R | GGAGTACGGCAAAGACCTCTTC |
| purD | PurD-F | CCGGACAGAAGGGAAGAGTTT |
|  | PurD-R | GTCACGTAGCGTTTGAGAACGT |
| purE | PurE-F | TGATCTCCCGGTCCGACTT |
|  | PurE-R | TGCTTGCTGTCGGGTAGCT |
| purF1 | PurF1-F | GCAAAAACATCTCGGCATCA |
|  | PurF1-R | CGGTCAAGGAACAGCTTGGA |
| purF2 | PurF2-F | AAATGGCCCCCGTAAACG |
|  | PurF2-R | GATGGTGTCGAAAGTGCTGTGA |
| purG | PurG-F | CGTGTTTGGGTGGGAGACTT |
|  | PurG-R | ACCTTTTTGGTGTTTCGATCGT |

**Table S4.** The primers involved in pyrimidine nucleosides biosynthesis used for real-time PCR

| Genes | Primers | Sequence (5’-3’) |
| --- | --- | --- |
| pyrA | PyrA-F | GGCGTGCCCCTTTTCAT |
|  | PyrA-R | GCAGCTTCTCGCTCATGCA |
| pyrB | PyrB-F | CATGTCCATGATGAAGCTCCTGTA |
|  | PyrB-R | AGGTGAGCGCCAAAAACG |
| pyrC1 | PyrC1-F | CGTCGCTTGCCAATGCT |
|  | PyrC1-R | GACGTCTTGACCTCGTAGGGATA |
| pyrC2 | PyrC2-F | TCGTGGCAGGCATTGTTG |
|  | PyrC2-R | GTCTGCAGCAGGGAGCTTCT |
| pyrD | PyrD-F | GCGACACCACGGACAAGAT |
|  | PyrD-R | GCCCAGCGGTTCCAAAA |
| pyrE | PyrE-F | TGATGACCAGCACGCTAAAAA |
|  | PyrE-R | TCCACAACGCCTCGACTCA |
| pyrF | PyrF-F | CTACTCGCCGCATGTGCTTA |
|  | PyrF-R | TTCCGATGTCAAGGAGCTCAA |
| pyrG | PyrG-F | TACGGCGCGAACAAGCA |
|  | PyrG-R | CAAAGCTCTGCGTGATGAAGTCT |
| pyrH | PyrH-F | CGCCCAGAAGGAGATCAAAC |
|  | PyrH-R | TGCCGGAGGAACGGTAGTT |
| pyrI | PyrI-F | TAGGCATTCCCCGAGTCATC |
|  | PyrI-R | CTTGGCATTTCCCACTGCTT |
| pyrJ | PyrJ-F | ACCCCTGCGTCACCTTTGT |
|  | PyrJ-R | GTGCTCTGAATCCGGATGGT |
| pyrK | PyrK-F | GGCCACATCGGAGGAGTTT |
|  | PyrK-R | CCTTCAGGCATGAAAATGATAAGG |
| pyrL | PyrL-F | CGCGGATCCGAATTCG |
|  | PyrL-R | TGATCCACCCCTCCAAGGA |

**Table S5. ORF analysis of functional genes involved in biosynthetic pathway of purine nucleotides in *H. sinensis***

| Enzymes | Location of genes | ORFs | Length (bp) | GenBank accession numbers |
| --- | --- | --- | --- | --- |
| punA | Unigene14697_All | purA | 855 | GCQL01014666 |
| Adenosine kinase | Unigene1297_All | purB | 326 | GCQL01001297 |
| APRT | Unigene12674_All | purC | 371 | GCQL01012671 |
| AMP deaminase | Unigene13557_All | purD1 | 378 | GCQL01013554 |
|  | Unigene3652_All | purD2 | 1032 | GCQL01003651 |
| IMP dehydrogenase | Unigene6288_All | purE1 | 407 | GCQL01006286 |
|  | Unigene9455_All | purE2 | 1047 | GCQL01009452 |
| GMP synthase | Unigene11031_All | purF1 | 331 | GCQL01011028 |
|  | Unigene11950_All | purF2 | 553 | GCQL01011947 |
|  | Unigene13696_All | purF3 | 490 | GCQL01013693 |
| Guanine deaminase | Unigene7330_All | purG1 | 328 | GCQL01007327 |
|  | Unigene9274_All | purG2 | 753 | GCQL01009271 |

**Table S6. ORF analysis of functional genes involved in biosynthetic pathway of pyrimidine nucleotides in *H. sinensis***

| Enzymes | Location of genes | ORFs | Length (bp) | GenBank accession numbers |
| --- | --- | --- | --- | --- |
| Dihydroorotase | Unigene8547_All | pyrA1 | 2718 | GCQL01008544 |
|  | Unigene8750_All | pyrA2 | 624 | GCQL01008747 |
|  | Unigene11749_All | pyrA3 | 984 | GCQL01011746 |
|  | Unigene8448_All | pyrA4 | 1095 | GCQL01008445 |
| Dihydroorotate dehydrogenase | Unigene13687_All | pyrB1 | 303 | GCQL01013684 |
|  | Unigene6100_All | pyrB2 | 660 | GCQL01006098 |
| Uridine monophosphate synthetase | Unigene3048_All | pyrC1 | 414 | GCQL01003047 |
|  | Unigene3106_All | pyrC2 | 768 | GCQL01003105 |
|  | Unigene3256_All | pyrC3 | 738 | GCQL01003255 |
| 5'-nucleotidase | Unigene10391_All | pyrD1 | 519 | GCQL01010388 |
|  | Unigene11624_All | pyrD2 | 1971 | GCQL01011621 |
| Uridine nucleosidase | Unigene17827_All | pyrE | 1281 | GCQL01017603 |
| udk | Unigene7973_All | pyrF | 300 | GCQL01007970 |
| UMP-CMP kinase | Unigene11229_All | pyrG1 | 1266 | GCQL01011226 |
|  | Unigene11252_All | pyrG2 | 912 | GCQL01011249 |
| ndk | Unigene17829_All | pyrH | 216 | GCQL01017605 |
| Apyras | Unigene1156_All | pyrI | 2049 | GCQL01001156 |
| ITPA | Unigene5342_All | pyrJ | 168 | GCQL01005340 |
| CTP synthase | Unigene6007_All | pyrK | 741 | GCQL01006005 |
| cdd | Unigene11624_All | pyrL | 1971 | GCQL01011621 |

**Figure S1.** The annotated purine metabolism pathway in *H. sinensis*. The red marks stand for the corresponding enzymes were annotated in *H. sinensis* annotation dataset.

**Figure S2.** The annotated pyrimidine metabolism pathway in *H. sinensis*. The red marks stand for the corresponding enzymes were annotated in *H. sinensis* annotation dataset.

**Figure S3.** Agarose gel electrophoresis of PCR products in biosynthetic pathway of purine nucleotides. Line A stands for purA, lane B1 stands for purB, lane C stands for purC, lane D1 stands for purD1, lane D2 stands for purD2, lane E1 stands for purE1, lane E2 stands for purE2, lane F1 stands for purF1, lane F2 stands for purF2, lane F3 stands for purF3, lane G1 stands for purG1, and lane G2 stands for purG2.

**Figure S4.** Agarose gel electrophoresis of PCR products in biosynthetic pathway of pyrimidine nucleotides. Lane A1 stands for pyrA1, lane A2 stands for pyrA2, lane A3 stands for pyrA3, lane A4 stands for pyrA4, lane B1 stands for pyrB1, lane B2 stands for pyrB2, lane C1 stands for pyrC1, lane C2 stands for pyrC2, lane C3 stands for pyrC3, lane D1 stands for pyrD1, lane D2 stands for pyrD2, lane E stands for pyrE, lane F stands for pyrF, lane G1 stands for pyrG1, lane G2 stands for pyrG2, lane H stands for pyrH, lane I stands for pyrI, lane J stands for pyrJ, lane K stands for pyrK, and lane L stands for pyrL.

**Figure S5.** Differential expression analysis of other four functional genes involved in biosynthetic pathway of purine nucleotides.

**Figure S6.** Differential expression analysis of other nine functional genes involved in biosynthetic pathway of pyrimidine nucleosides.
